# MSC-derived extracellular vesicle-associated miRNAs in CNS diseases: mechanisms of microglial polarization and therapeutic implications

**DOI:** 10.1186/s13287-026-05198-9

**Published:** 2026-07-29

**Authors:** Ye Chen, Fajuan Tang, Xihong Li

**Affiliations:** 1 Department of Emergency Medicine, West China Second University Hospital, Sichuan University, Chengdu, 610041 China; 2 Key Laboratory of Birth Defects and Related Diseases of Women and Children (Sichuan University), Ministry of Education, Chengdu, 610041 China

**Keywords:** Mesenchymal stem cells, Extracellular vesicles, Small extracellular vesicles, microRNAs, Microglial polarization, Neuroinflammation, Central nervous system diseases

## Abstract

Microglia-mediated neuroinflammation contributes to the pathogenesis of many central nervous system (CNS) diseases. After injury or infection, microglia can adopt pro-inflammatory programs that amplify neuronal damage. By contrast, reparative microglial states support inflammatory resolution, tissue repair and regeneration. Strategies that redirect harmful microglial activation toward reparative programs therefore have therapeutic relevance. Mesenchymal stem cell-derived extracellular vesicles (MSC-EVs), particularly small EV-enriched preparations, are increasingly studied as low-immunogenicity carriers of regulatory cargo. EV-associated microRNAs (miRNAs) are among the major payloads implicated in immunomodulation. This review synthesizes how MSC-EV-associated miRNAs regulate microglial polarization and neuroinflammation through TLR/NF-κB, MAPK, JAK/STAT, PI3K/Akt and Wnt/β-catenin signaling. We discuss evidence from ischemic stroke, traumatic brain injury, spinal cord injury, Alzheimer’s disease and sepsis-associated encephalopathy, and highlight EV terminology, donor source, species context and translational comparability as key barriers for future development.

## Mesenchymal stem cells and extracellular vesicles

### Overview of MSCs and MSC-derived EVs

Mesenchymal stem cells (MSCs) are adult mesoderm-derived stromal cells with self-renewal and multilineage differentiation capacity [1]. They can be isolated from bone marrow, adipose tissue, umbilical cord and other tissues, and are widely studied for regenerative and immunomodulatory applications [2]. Increasing evidence indicates that many MSC-mediated effects arise from paracrine signaling rather than direct engraftment, with secreted EVs acting as important mediators [3].

**Fig. 1:**
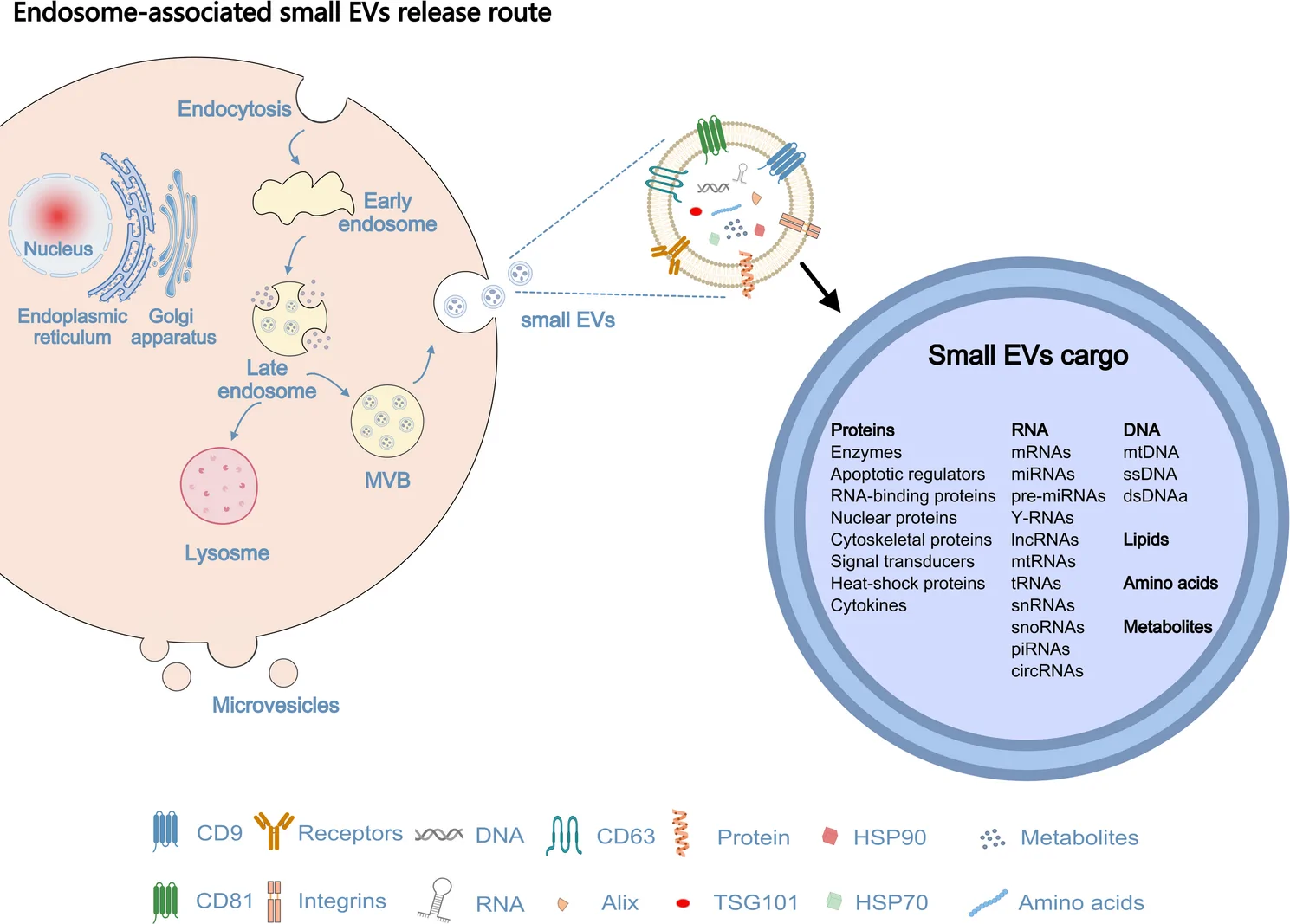
Generalized schematic of small extracellular vesicle biogenesis and representative cargo composition. **A** Biogenesis. The illustration summarizes an endosome-associated route in which intraluminal vesicles form within multivesicular bodies and are released after fusion with the plasma membrane. Because many MSC-EV studies do not definitively assign vesicles to a specific biogenesis pathway, this figure should be interpreted as an illustrative small-EV framework rather than evidence that all reviewed preparations are exclusively exosomes. **B** Structure and composition. Small EV-enriched preparations contain a lipid bilayer and commonly reported EV-associated proteins, lipids, metabolites and nucleic acids, including miRNAs. The markers and cargoes shown are representative and should be interpreted alongside the operational EV nomenclature used in this review

Small EVs are membrane-bound vesicles commonly recovered in nanoscale fractions and may arise through endosome-associated or other release routes [4]. They can transport nucleic acids, including DNA, mRNA and miRNAs, as well as proteins, lipids and metabolites. By transferring these cargoes between cells, EVs regulate diverse physiological and pathological processes (Fig. 1).

MSC-derived EVs (MSC-EVs) can cross the blood-brain barrier (BBB), show low immunogenicity and exhibit favorable biocompatibility [5]. Compared with cell transplantation, EV-based approaches offer advantages in preparation, storage, transport and targeted delivery [6–8]. Within the central nervous system (CNS), EVs are preferentially internalized by microglia, supporting their relevance to neuroinflammatory regulation [9]. MSC-EVs may promote neural repair by attenuating neuroinflammation, modulating microglial polarization, reducing neuronal apoptosis and influencing other glial populations [10–13]. They can also improve tissue structure and function by suppressing oxidative stress and apoptosis, regulating inflammatory responses and promoting angiogenesis [14]. In addition, MSC-EVs participate in crosstalk with T cells, B cells and NK cells [15], and reshape the immune microenvironment by modulating cytokines such as interleukins, tumour necrosis factor-alpha (TNF-α) and transforming growth factor-beta (TGF-β) [16, 17]. Among EV cargoes, miRNAs are particularly important functional mediators. These endogenous 19–25 nucleotide non-coding RNAs regulate target mRNAs through post-transcriptional repression or degradation. Through EV-mediated transfer, miRNAs can influence proliferation, differentiation and apoptosis in recipient cells [18]. Their stability in body fluids and association with disease states also make them candidate diagnostic and prognostic biomarkers [19]. In MSC-EVs, miRNAs are key effectors of neuroinflammatory suppression and microglial-state regulation [20, 21], providing a mechanistic basis for emerging cell-free therapeutic strategies.

### Operational terminology for MSC-derived EVs

In this review, MSC-EVs is used as an umbrella term for vesicle preparations released by mesenchymal stem/stromal cells. We use small EVs when cited studies report vesicles in nanoscale fractions but do not provide definitive evidence of endosomal biogenesis [22]. The term exosome is retained only when it reflects the wording of the original study or when the isolation and characterization strategy supports a biogenesis-based interpretation. This approach follows current ISEV guidance, which cautions against treating sEV and exosome as interchangeable terms and emphasizes transparent reporting of separation and characterization criteria [22].

### Source and species considerations for MSC-EVs

EV source is not a minor technical descriptor; it is a determinant of biological activity. MSC tissue origin, donor state, culture conditions and upstream processing can reshape EV yield, surface composition, RNA/protein cargo and functional potency [23]. Direct comparisons suggest that human BM-, AD- and UC-MSC-derived EV preparations are not interchangeable. Proteomic analyses have linked BM-MSC-EVs to regenerative signatures, AD-MSC-EVs to immunoregulatory features and UC-MSC-EVs to tissue-repair-associated profiles [24]. BM- and AD-MSC-derived EVs also differ in miRNA and tRNA content, further supporting source-specific interpretation of miRNA-mediated effects [13].

Species origin requires similar caution. Many CNS disease studies administer human MSC-EVs to rodent models, whereas others use rodent MSC-EVs or do not clearly report donor species. Cross-species designs are useful for preclinical screening, but they can complicate interpretation because cargo conservation, recipient-cell uptake, immune recognition and baseline microglial responses may differ between human and rodent systems. Species-specific variation in microglial biology may also limit direct translation from rodent models to human neuroinflammatory disease [25]. Future MSC-EV studies should therefore report donor species, MSC tissue source, culture conditions, EV characterization strategy, recipient model species and potency assays linked to microglial phenotypes.

## Microglia and neuroinflammation

Microglia are the resident immune cells of the CNS, originating from myeloid progenitor cells in the embryonic yolk sac, and they account for approximately 5%–15% of the total CNS cell population [26, 27]. They demonstrate wide CNS distribution, with particular enrichment in the hippocampus and substantia nigra [28]. Under physiological homeostasis, resting (M0) microglia exhibit a highly ramified morphology, continually performing immune surveillance via their processes [29, 30], and maintaining CNS homeostasis through cellular debris phagocytosis and synaptic remodeling regulation [27, 31].

Upon CNS injury or pathogen invasion, resting microglia are rapidly activated. Following the recognition of pathogen-associated molecular patterns (PAMPs) or damage-associated molecular patterns (DAMPs) by surface pattern recognition receptors (PRRs), they undergo a morphological transformation from a ramified to an amoeboid state and polarize toward specific functional phenotypes [32]. Microglial polarization is primarily categorized into M1 and M2 phenotypes, with distinct roles in neuroinflammation [33, 34].

The M1 phenotype is predominantly elicited by the Toll-like receptor 4/nuclear factor kappa-B (TLR4/NF-κB) signaling pathway following activation by lipopolysaccharides (LPS) or interferon-gamma (IFN-γ). This state results in the copious production of iNOS, ROS, and cytokines, such as TNF-α, IL-1β, IL-6, and IL-18. While fundamental for clearing pathogens, excessive M1 activity acts as a catalyst for neurotoxic damage [35].

Conversely, the transition to the M2 state is governed by the Janus kinase/signal transducer and activator of the transcription 6 (JAK/STAT6) pathway, triggered by IL-4 and IL-13. By upregulating arginase-1 (ARG-1) alongside protective factors such as insulin-like growth factor 1 (IGF-1), IL-10, and TGF-β, M2 microglia facilitate anti-inflammatory processes and neural repair [36, 37]. M1/M2 polarization is a complex dynamic process coregulated by the microenvironment, cellular metabolism, and transcriptional networks [38].

Microglial phenotypic polarization is tightly coupled with pyroptosis, collectively driving neuroinflammation [39]. M1 polarization serves as a critical upstream event for pyroptosis. It “primes” the transcription of the NLR family pyrin domain containing 3 (NLRP3) inflammasome and precursor interleukin-1 beta (pro-IL-1β) via the NF-κB pathway [40], and subsequently activates NLRP3 and Caspase-1 in response to a second signal (e.g., ATP). Activated caspase-1 cleaves gasdermin D (GSDMD), after which the GSDMD-N-terminal fragment forms pores, inducing pyroptosis and the release of inflammatory mediators [41]. In contrast, M2 polarization negatively regulates this process by inhibiting NF-κB activity.

In summary, M1 polarization provides the molecular basis for pyroptosis, while the inflammatory mediators released by pyroptosis amplify M1-type neuroinflammation, forming a vicious cycle that drives neuronal injury. Therefore, targeting the inhibition of excessive microglial M1 polarization represents a promising therapeutic strategy for blocking pyroptosis-associated neuroinflammation.

## Major signaling pathways modulated by miRNAs in MSC-EVs

### TLR/NF-κB

Within the CNS, macrophages, astrocytes, and microglia ubiquitously express TLR-4, a pivotal member of the PRR superfamily that governs apoptotic and inflammatory processes [42]. The receptor is tuned to detect specific molecular signatures: PAMPs such as Gram-negative LPS, and DAMPs including calcium-binding S100A8/9 proteins and high-mobility group box 1 (HMGB1) [43].

Under inflammatory stress, TLR-4 ligation catalyzes a myeloid differentiation primary response 88 (MyD88)-dependent response that engages the κB kinase (IKK) complex. This interaction forces the degradation of the inhibitory protein IκBα, thereby liberating NF-κB for nuclear translocation and subsequent gene expression [44]. As a pivotal transcription factor, NF-κB not only serves as the central driver of microglial activation but also regulates the phenotypic hallmarks of pro-inflammatory M1 microglia, thereby playing an essential role in maintaining the microglial M1/M2 phenotypic balance [45]. Since this cascade acts as a master regulator of M1-type inflammation, pharmacological blockade of the TLR-4/NF-κB sequence represents a compelling approach to ameliorate tissue damage in the CNS [46].

MSC-EVs and their encapsulated miRNAs exert multitarget, multilevel synergistic regulatory effects that collectively suppress this signaling pathway. At the receptor level, miR-182 directly targets TLR-4 to block signal initiation at its origin [47]. At the adaptor interface, signal propagation is halted by miR-124-3p, which intercepts tumor necrosis factor receptor-associated factor 6 (TRAF6) to prevent nuclear signaling [48]. Furthermore, miR-216b-5p facilitates the M1 to M2 phenotype transition. This miRNA functions by dual-targeting, inhibiting the TLR-4/NF-κB sequence while upregulating the PI3K/Akt pathway [49]. In a comparable manner, macrophage polarization is directed toward the M2 profile by miR-146a-5p, which targets interleukin-1 receptor-associated kinase 1 (IRAK1) and TRAF6 to negatively regulate NF-κB output [50, 51]. Thus, through the synergistic delivery of these bioactive components, MSC-EVs effectively dismantle the TLR-4/NF-κB inflammatory axis. This multitarget regulatory strategy not only reduces pro-inflammatory cell activation and cytokine release but also promotes microglial M2 polarization, offering novel therapeutic perspectives for CNS-related disorders.

### Mitogen-activated protein kinase (MAPK)

The mitogen-activated protein kinase (MAPK) signaling pathway constitutes a critical intracellular signaling cascade that mediates neuroinflammatory responses. This pathway encompasses several major branches, including ERK, p38, and JNK. Aberrant signaling—especially via the p38 and JNK axes—fuels microglial hyperactivity and cytokine storms that accelerate neuronal damage and demyelination [52, 53].

A defining therapeutic attribute of MSC-EVs is their ability to blunt the phosphorylation of pivotal MAPK transducers. Substantial data reveal that these EVs substantially reduce p-p38 and p-JNK levels in microglia, thereby curbing the output of ROS and key inflammatory agents such as TNF-α and IL-1β [54]. This suppression drives a phenotypic shift. It forces microglia out of the M1 state and into the restorative M2 lineage, fostering a neuroprotective milieu. Mechanistically, miRNAs such as miR-467f, miR-466q, and miR-532-3p execute this blockade by targeting upstream kinases, such as apoptosis signal-regulating kinase 1 (ASK1), effectively silencing the inflammatory cascade [55]. Moreover, miR-146a-5p can alleviate hemoglobin-induced microglial inflammation by modulating the TLR4/IRAK1/TRAF6/MAPK signaling axis [56]. Collectively, MSC-EVs impose precise, multitarget regulation over the MAPK signaling pathway, thereby effectively suppressing neuroinflammation and promoting CNS repair [57]. These findings underscore the substantial therapeutic promise of MSC-EVs as modulators of MAPK-driven neuroinflammatory processes.

### JAK/STAT

The JAK/STAT pathway is a critical signal transduction cascade that mediates cellular responses to cytokines and other extracellular signals and directly regulates gene expression [58, 59]. The canonical signaling sequence begins with cytokine–receptor ligation, which triggers JAK kinase recruitment and activation. Once phosphorylated, STAT proteins dimerize and enter the nucleus to drive transcriptional programs [58]. Within the CNS, the aberrant activity of this pathway fuels glial reactivity and neuroinflammation [60]. Specifically, the JAK2–STAT3 axis acts as a potent driver of neuroinflammatory states; in vitro data confirm its pivotal role in priming microglial activation and upregulating pro-inflammatory gene output [61]. However, the role of this pathway in microglial polarization is complex and context-dependent. It contributes not only to M1-type pro-inflammatory regulation but also to M2-type anti-inflammatory responses. For instance, LPS suppresses JAK1/STAT3 pathway activation during microglial M1 polarization, whereas MSC-EVs reverse this effect by enhancing JAK1/STAT3 phosphorylation [62]. Activation of the JAK1/3–STAT3/6 signaling cascade can drive microglia toward an M2 phenotype, accompanied by the release of anti-inflammatory cytokines (e.g., IL-10) and growth factors (e.g., TGF-β) [62].

Multiple miRNAs can target STAT3, a key regulatory node within this pathway. For instance, miR-124-3p can directly target and suppress STAT3, thereby effectively inhibiting M1-type microglial polarization [63]. Similarly, the suppression of STAT3 phosphorylation is mediated by miR-21a-5p, a process that lowers the output of inflammatory agents (specifically IL-1β and TNF-α) while favoring M2 microglial polarization and enhanced neuroprotection [64]. In summary, through the delivery of bioactive molecules, including miRNAs, MSC-EVs achieve precise bidirectional regulation of the JAK/STAT pathway. This mode of action is fundamental to their ability to attenuate neuroinflammatory cascades and support CNS repair, confirming their potential in clinical applications.

### PI3K/Akt

MSC-EVs exert neuroprotective, anti-inflammatory, and pro-regenerative effects within the CNS largely by modulating the PI3K/Akt signaling pathway [65]. This pathway represents a central regulatory node governing cellular metabolism, survival, inflammatory responses, and microglial phenotypic polarization [66, 67].

Phosphatase and tensin homolog (PTEN), an intrinsic negative regulator of PI3K/Akt signaling, plays a pivotal role in constraining pathway activation. miR-21a-5p enriched in MSC-EVs directly targets and suppresses PTEN, thereby releasing its inhibitory constraint on PI3K/Akt signaling. This pathway results in robust pathway activation and drives microglia toward an anti-inflammatory, reparative M2 phenotype [68, 69]. Activated Akt subsequently phosphorylates the downstream effector mTOR, a process that not only maintains autophagic homeostasis but also mitigates glutamate-induced excitotoxicity and Aβ-mediated neuronal injury [65].

Importantly, MSC-EVs also orchestrate interpathway crosstalk. For instance, miR-182 simultaneously suppresses the TLR4/NF-κB pathway—which promotes M1 polarization—while cooperating to activate the PI3K/Akt pathway to enhance M2 polarization [47]. By coupling the M1 blockade with M2 activation, this mechanism ensures a unidirectional transition in microglial identity. Ultimately, these results demonstrate that MSC-EVs do not merely dampen inflammation but serve as sophisticated modulators via their miRNA cargo.

### Wnt/β-catenin

The Wnt/β-catenin axis acts as a central governor of embryonic development, cell migration, and proliferation, while playing a decisive role in neuroprotection. Molecular initiation occurs when Wnt ligands complex with Frizzled (FZD) and LRP5/6 receptors to activate Disheveled (DVL). This event suppresses the β-catenin destruction machinery (Axin, APC, CK1, and Glycogen synthase kinase 3 beta (GSK-3β)), allowing β-catenin to stabilize in the cytosol. Subsequently, β-catenin migrates to the nucleus and partners with TCF/LEF factors to induce gene transcription [70]. Crucially, engaging this pathway fosters an anti-inflammatory M2 microglial state, thereby facilitating CNS repair and dampening inflammation [71]. MSC-EVs can deliver miR-124-3p and miR-26a, which target and degrade GSK-3β [72, 73]. GSK-3β is a key kinase within the β-catenin phosphorylation-dependent destruction complex. Its downregulation results in stabilized accumulation of β-catenin in the cytoplasm and subsequent nuclear translocation, which, in turn, activates a series of downstream target genes with anti-apoptotic, pro-survival, and pro-regenerative functions [74].

Notably, MSC-EV-mediated regulation of the Wnt/β-catenin pathway exhibits a finely tuned bidirectional nature. While MSC-EVs can activate this pathway via cargoes such as miR-409-3p and miR-1246, thereby effectively promoting neurite outgrowth [75], they can suppress excessive activation of this pathway by delivering other miRNAs such as miR-194, hence mitigating associated neuroinflammatory responses [76, 77]. This bidirectional regulatory mechanism maintains a dynamic balance between neuronal regeneration and inflammation suppression, provides a favorable microenvironment for neurons, prevents damage-induced cascades of apoptosis, and ultimately facilitates the recovery of neurological function.

**Fig. 2:**
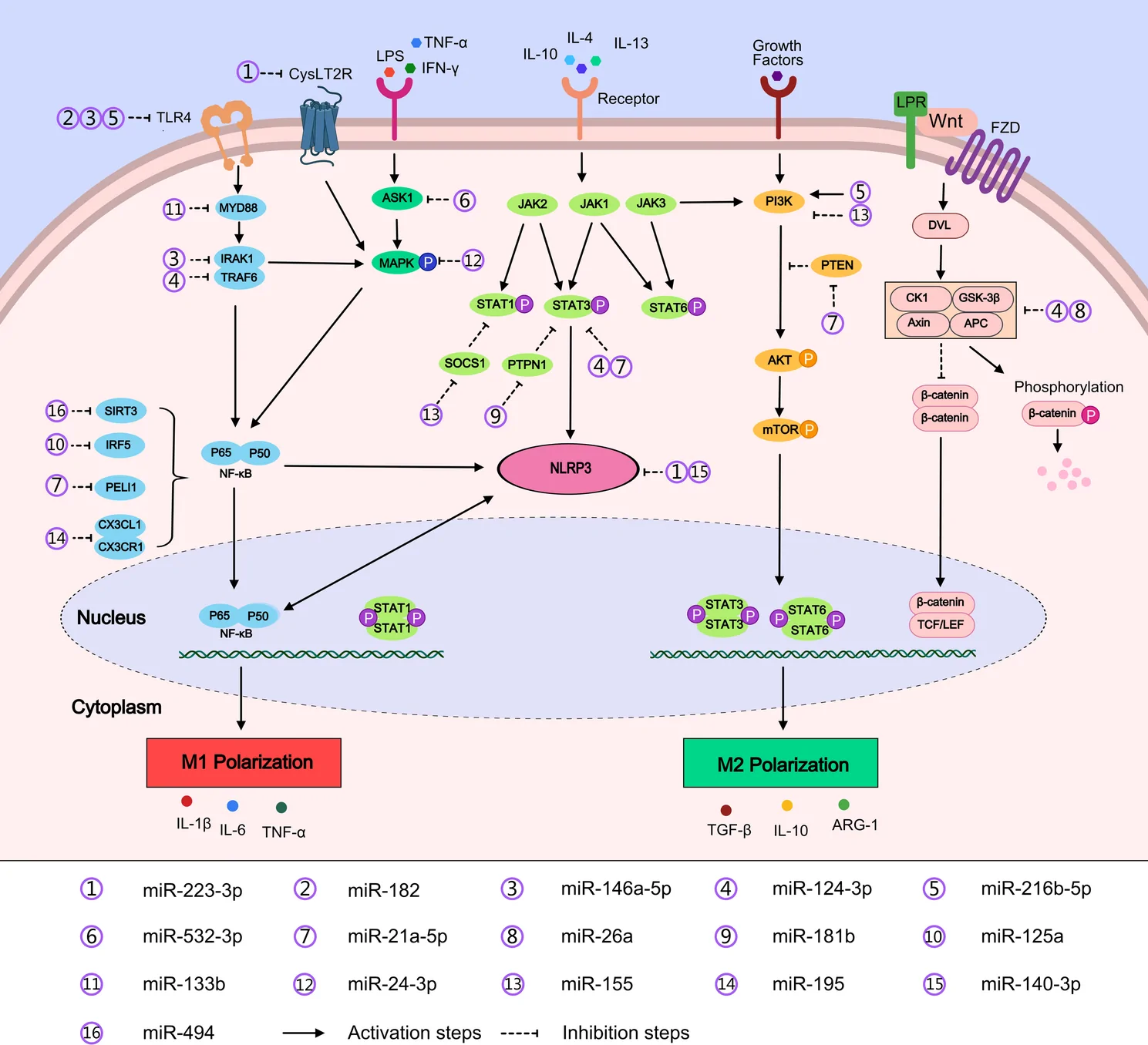
Simplified signaling map of representative MSC-EV-associated miRNAs that regulate microglial inflammatory states. The schematic highlights five recurring signaling axes discussed in the text: TLR/NF-κB, MAPK, JAK/STAT, PI3K/Akt and Wnt/β-catenin. Representative miRNAs are shown as pathway-level regulators rather than as an exhaustive target map. Detailed disease contexts and EV source information are summarized in Table 1

miRNAs highly enriched in MSC-EVs are key effector molecules in regulating microglial activity. The simultaneous governance of the NF-κB, MAPK, JAK/STAT, PI3K/Akt/mTOR, and Wnt/β-catenin axes underscores the robust therapeutic versatility of MSC-EVs. By exerting fine-tuned control over these networks, EVs function as precise rheostats that steer microglial fate, thereby reducing neuroinflammation and ensuring neuronal survival. Collectively, these mechanisms position MSC-EVs as a transformative strategy for neurological intervention (Fig. 2).

## The role of miRNAs within MSC-EVs in CNS diseases

### Ischemic stroke (IS)

IS is a prevalent cerebrovascular disease that affects the CNS. Although revascularization remains the standard of care, it frequently precipitates ischemia/reperfusion (I/R) injury, culminating in secondary brain damage via exacerbated oxidative stress and inflammatory responses [78]. Neuroinflammation constitutes a pivotal component of IS pathophysiology, characterized by microglial activation, peripheral immune cell infiltration, and excessive release of inflammatory mediators [79]. Consequently, modulating microglial polarization to facilitate a shift toward the M2 phenotype represents a highly promising therapeutic strategy for IS.

Against this background, the primary mechanism underlying the effective mitigation of post-IS neuroinflammation by MSC-EVs involves regulating microglial polarization and suppressing NLRP3 inflammasome activation [80, 81]. Mechanistically, MSC-EVs exert their therapeutic effects through several distinct pathways: (1) Inhibition of upstream signaling: MSC-EVs suppress the NF-κB signaling pathway and downregulate the transcription of NLRP3 and pro-IL-1β, thereby blocking the inflammasome “priming” step [82]; (2) direct interference with inflammasome function: MSC-EVs can directly inhibit activated NLRP3 inflammasomes, reducing the levels of IL-1β and IL-18 while upregulating anti-inflammatory cytokines such as IL-4 and IL-10, thus remodeling the immune microenvironment within the injury zone; and (3) delivery of regulatory miRNAs: MSC-EVs are enriched with specific miRNAs that directly target and silence NLRP3 expression, thereby inhibiting inflammasome activation and driving microglial polarization toward the M2 phenotype [83]. Therefore, a comprehensive understanding of the role of miRNAs in IS pathogenesis is essential for identifying novel therapeutic avenues.

#### miR-124

By delivering bioactive payloads to the injury site, BM-MSC-EVs mitigate I/R damage and support neurological recovery [84–86]. Among these cargoes, miR-124 stands out as a critical mediator of microglial plasticity [87], playing a broad role in neuronal survival and differentiation [88]. Although Dicer generates − 5p and − 3p strands, miR-124-3p functions as the primary effector. It acts by silencing the TLR4/NF-κB/NLRP3 inflammatory axis—a key driver of cytokine storms in IS—thereby attenuating the release of IL-1β and TNF-α [89, 90]. In parallel, miR-124 skews microglia toward the restorative M2 phenotype, significantly enhancing the secretion of protective factors such as IL-10 and Arg-1 [91]. This coordinated regulation effectively reverses the pathological M1/M2 balance [92].

#### miR-223-3p

Neuroprotection in IS is significantly enhanced by MSC-EVs, which utilize specific cargoes—specifically miR-223-3p—to tune microglial polarization [93]. This miRNA functions as a potent switch for microglial phenotypes. By exerting coordinated effects on downstream targets, it suppresses M1 pro-inflammatory activation and conversely promotes a shift toward the anti-inflammatory M2 phenotype.

The suppression of the M1 pro-inflammatory phenotype is primarily achieved by modulating the following signaling pathways:

1. Targeting the NLRP3 inflammasome: miR-223-3p has been confirmed to directly bind to the 3’ untranslated region (3’-UTR) of NLRP3, thereby inhibiting its transcription. This pathway consequently downregulates NLRP3, Caspase-1, and downstream IL-1β expression, effectively attenuating NLRP3 inflammasome activation and reducing the release of key pro-inflammatory cytokines [94, 95].
2. A key anti-inflammatory pathway involves the suppression of the Triggering Receptor Expressed on Myeloid cells 1 (TREM1) by human umbilical cord MSC-derived EVs (HUC-MSC-EVs). Relying on an HMGB1-dependent mechanism, this downregulation inhibits p38 MAPK signaling, thereby lowering TNF-α and IL-1β output [93].

In parallel, the transition toward the anti-inflammatory M2 phenotype is facilitated by miR-223-3p through its regulation of the cysteinyl leukotriene receptor 2 (CysLT2R)/extracellular signal-regulated kinase 1/2 (ERK1/2) signaling axis. Mechanistically, this miRNA counteracts the CysLT2R-mediated stimulation of ERK1/2 and MAPK cascades. Consequently, this blockade not only prevents M1 differentiation but also accelerates M2 conversion, serving to mitigate neuroinflammation and acute cerebral damage [96, 97].

#### miR-146a-5p

IRAK1 and TRAF6 act as critical upstream adaptor proteins within the NF-κB signaling pathway, playing a pivotal role in M1 microglia activation and polarization. miR-146a-5p specifically targets and suppresses the expression of IRAK1 and TRAF6 [98, 99]. By inhibiting these key proteins, miR-146a-5p effectively impedes NF-κB pathway activation, thereby significantly curtailing the production and release of downstream pro-inflammatory cytokines, including IL-6, TNF-α, and IL-1β. This suppression of the pro-inflammatory M1 pathway synergistically facilitates the transition of microglia toward the anti-inflammatory M2 phenotype. Through a complex regulatory network, miR-146a-5p indirectly enhances the expression of anti-inflammatory factors such as IL-10 and TGF-β1, which are signature cytokines of M2 microglia [100]. Consequently, MSC-EVs, via their cargo miR-146a-5p, precisely target the IRAK1/TRAF6/NF-κB signaling axis. This dual action—inhibiting M1 pro-inflammatory polarization while promoting the M2 anti-inflammatory shift—mitigates neuroinflammatory injury following IS [101].

### Traumatic brain injury (TBI)

TBI comprises primary mechanical injury and a subsequent secondary injury cascade. This secondary injury involves complex pathological processes, including neuroinflammation, excitotoxicity, and apoptosis, which ultimately exacerbate BBB disruption and neuronal death [102].

A cornerstone of MSC-EV therapy in TBI is the potent suppression of inflammatory cascades. Specifically, the NLRP3 inflammasome—a multiprotein complex markedly elevated in microglia post-injury—acts as a central hub for neuroinflammation and secondary damage [103, 104]. MSC-EVs intervene by arresting NLRP3 activation, effectively disrupting the downstream enzymatic machinery. This blockade prevents caspase-1 cleavage, thereby halting the maturation and subsequent secretion of key pro-inflammatory cytokines, IL-1β and IL-18 [105, 106]. Suppressing the NLRP3 pathway further attenuates the persistent activation of downstream inflammatory cascades, such as p38/MAPK, thereby curtailing the production of pro-inflammatory cytokines during the acute and chronic phases of TBI and improving neurological function [107]. Beyond inflammation suppression, MSC-EVs actively drive tissue repair. For instance, EVs derived from stem cells from human exfoliated deciduous teeth (SHED-EVs) facilitate the phenotypic shift of microglia to the restorative M2 state. Simultaneously, EVs from other sources, such as human umbilical cord MSCs (HUC-MSC-EVs), safeguard neurons against cell death by recalibrating the Bcl-2/Bax apoptotic ratio [108, 109].

In the context of TBI pathology, miRNAs serve as critical components of MSC-EVs, playing a key role in therapy through multitarget mechanisms that effectively mitigate post-TBI neuroinflammation and secondary injury.

#### miR-124-3p

The pathophysiological core of TBI lies in dysregulated neuroinflammation, a process crucially influenced by microglial phenotypic polarization [110]. Research indicates that MSC-EVs function as natural nanocarriers, delivering miR-124-3p to microglia within injured brain regions. This delivery induces a shift toward M2 polarization, thereby conferring robust neuroprotection [111].

Mechanistically, miR-124-3p exerts its effects through several pathways. Firstly, it blocks the amplification of pro-inflammatory signaling cascades at the upstream level by inhibiting the TLR4 pathway and the expression of its downstream adaptor TRAF6 [112, 113]. Secondly, it mitigates secondary injury in TBI by modulating multiple signaling axes. It inhibits mTOR signaling and phosphodiesterase 4B (PDE4B) expression [114], regulates the RELA/ApoE axis (specifically, inhibiting RELA to upregulate apolipoprotein E [ApoE]) [115], and targets the p38 MAPK/glutamate transporter 1 (GLT-1) axis (suppressing p38 MAPK to enhance GLT-1 upregulation, thereby reducing glutamate excitotoxicity) [53, 116]. Beyond immunomodulation, miR-124-3p directly safeguards neuronal integrity by intercepting specific injury cascades. Mechanistically, it suppresses autophagy mediated by the FAK family kinase-interacting protein of 200 kDa (FIP200). Simultaneously, it dampens the signaling axis involving Rho-associated coiled-coil containing protein kinases 1 and 2 (ROCK1/ROCK2), thereby preventing downstream apoptotic events [117, 118]. In TBI animal models, miR-124-3p treatment yields significant therapeutic benefits, including reduced lesion volume, improved neurological severity scores, and attenuated hippocampal neuronal loss. This functional recovery coincides with a distinct molecular shift: the suppression of pro-apoptotic Bax and inflammatory cytokines (notably TNF-α and IL-1β), paired with the simultaneous elevation of Bcl-2 and anti-inflammatory signals [119–121]. Crucially, this therapeutic approach promotes endogenous repair processes, such as angiogenesis and neurogenesis, laying the foundation for long-term functional recovery [122].

In conclusion, MSC-EVs, via the delivery of the key regulator miR-124-3p, modulate microglial polarization toward the M2 phenotype through multitarget and multi-pathway mechanisms. This pathway effectively ameliorates post-TBI neuroinflammation and facilitates neural repair, providing a solid theoretical basis and promising prospects for developing EV-based cell-free therapies for TBI [123, 124].

#### miR-21

In the pathological microenvironment of TBI, the expression and function of miR-21 exhibit significant complexity and duality [125]. Substantial evidence indicates that miR-21 plays a detrimental role by promoting inflammation and exacerbating injury post-TBI. In TBI models, EVs derived from injured neurons are enriched with miR-21-5p, which directly binds to and activates Toll-like receptor 7 (TLR7) on the endosomal membrane. This interaction triggers the NF-κB signaling pathway, driving microglial polarization toward the pro-inflammatory M1 phenotype [126]. Concurrently, miR-21 has been directly implicated in BBB disruption and TBI pathology exacerbation [127]. These findings corroborate clinical observations where elevated EV-associated miR-21 levels serve as diagnostic biomarkers for TBI, whereas reduced intracerebral levels correlate with favorable prognoses, including attenuated neuroinflammation, improved BBB integrity, and reduced neuronal apoptosis.

Conversely, when delivered as a therapeutic cargo via HUC-MSC-derived EVs, miR-21 demonstrates distinct neuroprotective and anti-inflammatory functions. Mechanistically, miR-21 functions as a dual regulator of microglial plasticity. First, it directly silences pro-inflammatory mediator programmed cell death 4 (PDCD4), an interaction that effectively halts NF-κB signaling [128]. Second, by targeting and suppressing PTEN, miR-21 disinhibits the PI3K/Akt axis. This molecular cascade is instrumental in driving the transition of microglia toward the restorative M2 lineage. HUC-MSC-EVs, which are naturally enriched with miR-21, promoted neurological recovery in TBI rat models and effectively suppressed microglial hyperproliferation, migration, and the release of inflammatory mediators [129].

This apparent paradox highlights the dual nature of the miR-21 function. Consequently, when evaluating miR-21 as a therapeutic target or biomarker for TBI, careful consideration of its cellular origin and delivery vehicle is imperative. This contextual understanding is crucial for elucidating its precise role within the complex regulatory network of neuroinflammation.

#### miR-181b

Cellular survival and inflammatory control after TBI are critically dependent on miR-181b activity. Experimental upregulation of this miRNA revealed blunt apoptotic and inflammatory cascades, functioning principally by steering microglial differentiation into the anti-inflammatory M2 state [130]. IL-10/STAT3 signaling pathway activation drives this process. As a potent anti-inflammatory cytokine, IL-10 binds to its receptor to activate downstream STAT3, which subsequently suppresses pro-inflammatory gene transcription and enhances M2-associated gene expression, thereby exerting significant neuroprotection [86].

In this context, BM-MSC-EVs serve as natural nanocarriers for miR-181b, facilitating its efficient internalization by microglia within the brain parenchyma. Mechanistically, miR-181b targets protein tyrosine phosphatase nonreceptor type 1 (PTPN1). Under normal conditions, PTPN1 acts as a negative regulator of the STAT3 pathway by dephosphorylating activated STAT3 (p-STAT3). By inhibiting PTPN1, miR-181b sustains IL-10/STAT3 signaling axis activation. This persistent STAT3 activation not only drives the M2 microglial shift but also establishes a positive feedback loop wherein M2 microglia secrete additional IL-10, further amplifying the anti-inflammatory effect [86]. Ultimately, this regulatory network, mediated by the MSC-EVs/miR-181b/IL-10/STAT3 axis, effectively curtails the release of pro-inflammatory cytokines (e.g., TNF-α and IL-6) and significantly mitigates post-TBI neuroinflammation, highlighting a novel molecular target for therapeutic strategies focused on modulating microglial polarization.

### Spinal cord injury (SCI)

SCI is a severe traumatic condition of the CNS characterized by a pathological progression involving primary and secondary injury mechanisms. Primary injury refers to immediate neuronal death and axonal disruption resulting directly from mechanical trauma. Crucially, this initial insult precipitates a complex cascade of secondary injury events, wherein the neuroinflammatory cascade serves as a central driver, leading to local edema, neuronal apoptosis, and reactive gliosis, thereby exacerbating the extent of the lesion [131]. Although current clinical interventions—comprising surgery, pharmacotherapy, and vasopressor support—offer a degree of neuroprotection during the acute phase, therapeutic strategies capable of fully reversing tissue damage or effectively promoting functional recovery in the chronic phase remain elusive. Neuroinflammation plays a decisive role in secondary SCI injury; as the resident immune cells of the CNS, microglial activation and polarization status are critical determinants in regulating the progression of neuroinflammation [132]. MSC-EVs have demonstrated immense potential in SCI therapeutics, attributed to their robust immunomodulatory, pro-angiogenic, and axon-regenerative capacities. Specifically, various miRNAs within MSC-EVs can modulate post-SCI neuronal apoptosis and inflammatory responses, paving the way for the development of novel therapeutic strategies.

#### miR-125a

Post-SCI microglial polarization is critically governed by miR-125a. BM-MSC-EVs transport miR-125a to the lesion site, effectively driving macrophages toward the reparative M2 phenotype [133]. At the molecular level, intracellular miR-125a binds to and silences interferon regulatory factor 5 (IRF5). Given the IRF5’s role as a master regulator of M1 pro-inflammatory commitment, its suppression effectively dismantles the M1 signaling axis. Consequently, this inhibition yields a marked reduction in pro-inflammatory cytokine profiles with a concomitant rise in M2-specific markers, such as Arg1. By restructuring the inflammatory milieu, this mechanism ameliorates SCI pathology, highlighting a viable therapeutic avenue. Consequently, by inhibiting IRF5, miR-125a significantly attenuates the levels of various pro-inflammatory cytokines while concurrently upregulating M2 phenotypic markers (e.g., Arg1). This process remodels the inflammatory microenvironment and mitigates the pathological progression of SCI, thereby presenting a promising therapeutic strategy for SCI treatment [133].

#### miR-133b

During the pathological progression of SCI, EVs derived from adipose-derived mesenchymal stem cells (AD-MSC-EVs) act as potent inhibitors of M1 microglial activation. These vesicles significantly dampen the release of pro-inflammatory cytokines, such as IL-1β, IL-2, and TNF-α, while concurrently augmenting the production of anti-inflammatory mediators like IL-10 and IL-13. This dual action facilitates a decisive phenotypic shift from the inflammatory M1 state to the reparative M2 state, thereby remodeling the lesion microenvironment to favor recovery [134]. AD-MSC-EV-derived miR-133b has been confirmed to target and silence genes such as RhoA, consequently modulating microglial proliferation, migration, and inflammatory responses [135]. In the pathological microenvironment of SCI, M1 microglial polarization is typically driven by signaling pathways such as NF-κB, resulting in the excessive release of pro-inflammatory mediators, including iNOS, TNF-α, and IL-1β [136]. Upon delivery into the cytoplasm, miR-133b specifically binds to MyD88. This interaction promotes the degradation or translational suppression of MyD88 mRNA, thereby impeding M1 polarization and alleviating neuroinflammation. Fundamentally, the redirection of microglial polarization—away from the inflammatory M1 state and toward the reparative M2 phenotype—fosters a microenvironment conducive to neuronal viability and axonal regrowth. This remodeling is instrumental in facilitating functional recovery [131].

#### miR-124-3p

In the pathological progression of SCI, BM-MSC-EV-derived miR-124-3p exerts therapeutic effects through distinct pathways. It mitigates spinal cord ischemia-reperfusion injury by targeting endoplasmic reticulum to nucleus signaling 1 (ERN1), thereby promoting macrophage polarization toward the anti-inflammatory M2 state [137]. Furthermore, this miRNA facilitates the transition of microglia toward the M2 phenotype, a shift that is indispensable for tissue repair and neuroregeneration [138].

Beyond modulating microglial polarization, miR-124-3p confers neuroprotection via multiple additional mechanisms, such as inhibiting the activation of neurotoxic astrocytes to improve the injury microenvironment [139]. Regarding the regulation of pyroptosis, EVs primed with resveratrol have been shown to upregulate miR-124-3p. This upregulation targets and inhibits death-associated protein kinase 1 (DAPK1), subsequently blocking the NLRP3/Caspase-1/GSDMD-mediated pyroptosis pathway and significantly attenuating neuroinflammation in SCI [140].

#### miR-24-3p

miR-24-3p modulates the pathological progression of SCI via at least two distinct signaling pathways. BM-MSC-EV-derived miR-24-3p directly targets and inhibits MAPK9 [141]. As MAPK9 serves as an upstream kinase of the JNK signaling pathway, its inhibition effectively abrogates the downstream JNK/c-Jun/c-Fos signaling cascade. Given that this axis acts as a critical driver of apoptosis and inflammation, this mechanism enables miR-24-3p to significantly attenuate post-SCI neuronal death and neuroinflammation [141].

As a secondary mechanism, miR-24-3p attenuates microglial inflammatory responses via the direct modulation of mitogen-activated protein kinase-activated protein kinase 2 (MK2). Functioning as a critical downstream effector of the p38 MAPK signaling axis, MK2 governs the posttranscriptional stability of inflammatory cytokine mRNA. Consequently, the inhibition of MK2 by miR-24-3p obstructs M1 microglial polarization and the associated efflux of pro-inflammatory cytokines. This intervention effectively remodels the deleterious microenvironment, thereby mitigating the pathological severity of SCI [142].

#### miR-21a-5p

miR-21a-5p serves as a critical effector molecule enriched within BM-MSC-EVs, and its delivery has been demonstrated to effectively promote locomotor recovery following SCI [143].

The underlying mechanism acts primarily through the regulation of the Pellino E3 Ubiquitin Protein Ligase 1 (PELI1) signaling axis. Upon delivery to microglia by BM-MSC-EVs, miR-21a-5p directly targets and suppresses PELI1 expression. PELI1 downregulation facilitates microglial polarization toward the M2 phenotype, thereby effectively attenuating the neuroinflammatory response [144]. Furthermore, the modulation of this signaling axis potently inhibits microglial pyroptosis, consequently mitigating secondary injury and fostering neurological functional recovery post-SCI [145].

#### miR-216a-5p

miR-216a-5p represents a key therapeutic constituent of BM-MSC-EVs. In rat SCI models, the administration of BM-MSC EVs enriched with miR-216a-5p yields significantly superior outcomes compared to the direct transplantation of BM-MSCs, specifically in improving locomotor recovery, attenuating neuronal apoptosis, and suppressing inflammation mediated by M1 microglial polarization [70].

Mechanistically, therapeutic efficacy relies centrally on the robust suppression of the TLR4/NF-κB signaling axis, evidenced by a marked reduction in the expression profiles of TLR4, the adaptor protein MyD88, and NF-κB [146]. Additionally, miR-216a-5p functions by directly binding to and inhibiting high-mobility group box 1 (HMGB1), effectively intercepting HMGB1-mediated stimulation of the TLR4/NF-κB cascade [147]. Beyond this, the regulatory scope of miR-216a-5p is multifaceted; it operates via a dual-pronged mechanism that concurrently attenuates TLR4/NF-κB signaling while activating the PI3K/AKT pathway. This synchronized modulation acts synergistically to propel the microglial phenotypic shift away from the pro-inflammatory M1 state and toward the anti-inflammatory M2 phenotype, ultimately amplifying the therapeutic outcome [49].

### Alzheimer’s disease (AD)

The pathogenesis of AD is fundamentally governed by the deposition of amyloid-β (Aβ) plaques—originating from the dysregulated proteolytic cleavage of amyloid precursor protein (APP)—and the formation of neurofibrillary tangles composed of microtubule-associated protein tau. These primary insults sequentially trigger a cascade of complex secondary damage [148]. Critically, the sustained bias of microglial polarization toward the pro-inflammatory M1 phenotype, occurring alongside a deficit in anti-inflammatory M2 functionality, serves as a pivotal catalyst for chronic neuroinflammation and neuronal attrition [149]. However, there is a lack of effective clinical therapeutic interventions capable of reversing or retarding disease progression.

MSC-EVs exert pleiotropic therapeutic effects in AD through the following mechanisms:

1. Regulation of microglial polarization and promotion of Aβ clearance: MSC-EVs significantly enhance the capacity of microglia to express Aβ-degrading enzymes—insulin-degrading enzyme (IDE) and neprilysin (NEP)—thereby effectively reducing both cerebral Aβ plaque burden and soluble Aβ levels [150].
2. Mitigation of oxidative stress and direct neuroprotection: MSC-EVs directly counteract Aβ-induced toxicity, significantly reducing reactive oxygen species (ROS) levels in hippocampal neurons of AD models [151].
3. Improvement of cognitive function: MSC-EVs treatment significantly delays the deterioration of cognitive deficits; these multidimensional neuroprotective effects ultimately culminate in marked improvements in cognitive function [152].

In the context of MSC-EVs-mediated AD therapy, cargo miRNAs exert a multidimensional, network-based regulatory function. These miRNAs act not only as effector molecules of neuroprotection by targeting and inhibiting key pathological pathways but also—depending on their expression context—may indirectly participate in or even drive pathological processes due to dysregulation [153, 154].

#### miR-146a

miR-146a functions as a pivotal regulator of neuroinflammation and microglial polarization in AD, exhibiting a dual nature that encompasses therapeutic potential and involvement in pathological progression.

First, miR-146a functions as a potent anti-inflammatory and neuroprotective factor. For instance, hippocampal miR-146a levels are typically reduced in AD mouse models. However, intracerebroventricular transplantation of BM-MSCs can significantly restore these levels, thereby attenuating neuroinflammation and improving cognitive function [155]. Research confirms that MSC-EVs engineered to overexpress miR-146a effectively mitigate AD-associated neuroinflammation, primarily by modulating the polarization state of microglia [156]. Additionally, in vitro studies indicate that small molecules, such as curcumin, suppress AD-related inflammatory responses via miR-146a upregulation [157].

Conversely, miR-146a is also implicated as a marker of pro-inflammatory status and disease progression. In both AD patients and animal models, miR-146a expression is frequently elevated, suggesting its utility as a potential biomarker for disease trajectory [158, 159]. On one hand, high miR-146a expression correlates with amyloid-β (Aβ) deposition and increased ROS levels, ultimately exacerbating cognitive impairment. On the other hand, the miR-146a gene is transcribed and processed into mature miR-146a-5p, which specifically targets and inhibits IRAK1 and TRAF6, leading to a significant reduction in MAPK activation [158]. Functionally, however, blocking miR-146a activity can yield therapeutic benefits. For example, miR-146a downregulation promoted neurite outgrowth and inhibited apoptosis, inflammatory responses, and STAT1/MYC pathway activation in AD cell models [160]. Ultimately, miR-146a’s contribution to AD pathology is intricate and highly situational. Its precise biological impact appears to be dictated by the intersection of disease progression, cellular specificity, and the signaling architecture of the local microenvironment.

#### miR-155

Notably, miR-155’s functional contribution to AD shows a distinct duality that depends strongly on the cellular context. Marked miR-155 upregulation in AD brains positions it as a key driver of microglial M1-phenotype polarization [161]. Mechanistically, miR-155 delivered by BM-MSC-EVs specifically binds to and represses the expression of suppressor of cytokine signaling 1 (SOCS1) and SH2-containing inositol 5’-phosphatase 1 (SHIP1). This suppression relieves the inhibition on the JAK2/STAT1 and PI3K/Akt–NF-κB signaling pathways, thereby driving microglial M1 polarization and amplifying neuroinflammation. This pro-inflammatory effect primarily operates through downstream target-gene regulation, which promotes the excessive production of pro-inflammatory cytokines—including TNF-α, IL-1β, and IL-6—consequently exacerbating neuroinflammation and cognitive impairment [162].

However, miR-155’s function in AD is far from being exclusively pro-inflammatory. Research indicates that while the microglia-specific knockout of miR-155 in AD mouse models effectively reduces amyloid-β (Aβ) plaque burden, it unexpectedly precipitates neuronal hyperexcitability and seizures [163]. These findings suggest that miR-155 plays an indispensable role in maintaining neuronal network homeostasis and that its indiscriminate inhibition may induce severe adverse effects. Further mechanistic investigations have elucidated this complexity: microglial miR-155’s deletion disrupts IFN-γ signaling, inducing a distinct activation state that paradoxically confers neuroprotection [164].

Therefore, while targeting miR-155 activity represents a potential therapeutic strategy for attenuating neuroinflammation, its critical function in preserving neuronal homeostasis warrants careful consideration.

#### miR-195

Notably, miR-195 exerts neuroprotective effects in AD pathophysiology via multitarget mechanisms, particularly by modulating amyloid-β (Aβ) biogenesis and microglial polarization. It directly targets and binds to the mRNA of β-site APP Cleaving Enzyme 1 (BACE1), thereby downregulating BACE1 protein expression at the posttranscriptional level. Given that BACE1 functions as the critical rate-limiting enzyme in the amyloidogenic processing of APP, its inhibition by miR-195 effectively curtails Aβ production, directly intervening in the core pathological mechanism of AD [165].

Moreover, miR-195 exerts regulatory authority over the C-X3-C Motif Chemokine Ligand 1 (CX3CL1)/C-X3-C Motif Chemokine Receptor 1 (CX3CR1) axis, a fundamental pathway mediating neuron–microglia crosstalk. By concurrently suppressing CX3CL1 and CX3CR1 expression, miR-195 modulates microglial activation and promotes a shift toward the anti-inflammatory M2 phenotype [166].

### Sepsis-associated encephalopathy (SAE)

SAE manifests as widespread cerebral impairment precipitated by systemic infection, presenting clinically with acute deficits in cognition, consciousness, and behavioral patterns. This condition constitutes a major factor underlying the elevated mortality observed in sepsis cohorts. Central to SAE pathophysiology is neuroinflammation, a cascade in which microglia—the resident immune sentinels of the CNS—occupy a critical regulatory position [167].

Microglia exert multifaceted regulatory control over SAE pathological progression. Activated M1 microglia produce and release copious amounts of pro-inflammatory mediators. These mediators not only directly mediate synaptic injury and neuronal death but also target cerebrovascular endothelial cells, increasing permeability and compromising BBB integrity [168, 169]. A compromised BBB subsequently permits the influx of peripheral inflammatory factors and immune cells into the CNS, establishing a vicious cycle that propels SAE pathology. Furthermore, various danger signals (e.g., ROS, bacterial toxins) trigger the assembly of the intracellular NLRP3 inflammasome within microglia. This pathway induces pyroptosis, thereby drastically amplifying the inflammatory cascade [170]. Concurrently, M1 microglia generate excessive ROS, which induces neuronal mitochondrial dysfunction. This results in bioenergetic failure and the disruption of intracellular calcium homeostasis, ultimately culminating in neuronal death [171].

Given the central position of microglia within the SAE pathological network, targeting their functional phenotype—specifically, suppressing excessive polarization toward the deleterious M1 phenotype—has emerged as a highly promising therapeutic strategy [172].

#### miR-140-3p

Within the therapeutic cargo of MSC-EVs, miR-140-3p occupies a critical position, governing SAE pathology by modulating microglia-mediated neuroinflammation and pyroptosis. High mobility group box 1 (HMGB1), a prominent damage-associated molecular pattern (DAMP), acts as a master regulator of neuroinflammation, synaptic degeneration, and cognitive dysfunction in SAE [173, 174]. In murine SAE models, miR-140-3p potently suppresses hippocampal HMGB1 expression. This blockade effectively curtails downstream inflammatory cascades, a finding corroborated by significant reductions in major pro-inflammatory cytokines (IL-1β, IL-6, TNF-α) and M1 microglial markers [172]. Additionally, miR-140-3p markedly dampens the expression of NLRP3, Caspase-1, and the pore-forming executioner GSDMD-N (the N-terminal domain of gasdermin D). Consequently, the inhibition of this axis effectively abolishes microglial pyroptosis [172]. This therapeutic strategy encompasses the regulation of cellular metabolism.

EVs enriched with miR-140-3p can upregulate the levels of S-lactoylglutathione, a neuroprotective metabolite, in the hippocampi of SAE mice. In vitro assays further confirm that S-lactoylglutathione alone is sufficient to inhibit microglial inflammation and pyroptosis [172]. In conclusion, the MSC-EVs-mediated delivery of miR-140-3p specifically targets HMGB1 to synergistically modulate neuroinflammation, pyroptosis, and protective metabolic pathways. This approach effectively improves survival rates in SAE mice, highlighting a highly promising novel strategy for SAE intervention.

#### miR-494

In the context of SAE-induced neuroinflammation, miR-494 can modulate microglial activation by specifically targeting Sirtuin 3 (SIRT3), thereby ameliorating SAE pathology [175].

SIRT3, a mitochondrial deacetylase, acts as a pivotal regulatory node in microglial activation [176]. In the neuroinflammatory milieu characteristic of SAE, the aberrant upregulation of SIRT3 expression or activity acts as a potent driver of microglial polarization toward the pro-inflammatory M1 phenotype. This shift precipitates a massive release of inflammatory mediators, thereby exacerbating neurotoxicity. At the molecular level, miR-494 selectively targets the 3’-UTR of SIRT3 mRNA to induce translational silencing, resulting in a distinct downregulation of SIRT3. This abrogation of SIRT3 function effectively halts downstream pro-inflammatory signaling, consequently checking microglial hyperactivation. Consequently, this inhibition facilitates a phenotypic shift from the M1 state to the anti-inflammatory M2 state, ultimately mitigating SAE-associated neuronal injury [175].

**Table 1:** The role of MSC-derived EV-associated miRNAs in CNS diseases

miRNA	Effect on microglial polarization	Target genes or signaling pathways	Associated diseases	MSC/EV source and species context	References
miR-124/ miR-124-3p	↓M1, ↑M2	IS: ↓NLRP3, ↓TLR4/NF-κB TBI: 1.↓TLR4, ↓TRAF6, ↓mTOR, ↓PDE4B, ↓Rela, ↑ApoE. 2.↓p38 MAPK, ↑GLT-1. 3.↓FIP200, ↓ROCK1/ROCK2. SCI: 1.↓ERN1. 2.↓MYH9, ↑PI3K/Akt, ↓NF-κB. 3.↓DAPK1, ↓NLRP3/Caspase-1/GSDMD.	IS TBI SCI	BM-MSC-EVs; mainly rodent IS/TBI/SCI models; source varies among cited studies.	[84–92] [110–124] [137–140]
miR-223-3p	↓M1, ↑M2	1.↓NLRP3 2.↓TREM1-p38 MAPK 3.↓CysLT2R-ERK1/2	IS	MSC-EVs, including HUC-MSC-EVs in the TREM1/p38 MAPK study; mainly rodent IS models.	[93–97]
miR-146a-5p	↓M1, ↑M2	↓IRAK1, ↓TRAF6, ↓NF-κB	IS	MSC-EVs; source and species are not consistently specified across cited studies.	[98–101]
miR-146a	M1/M2 (Dual function)	1.↓IRAK1, ↓TRAF6, ↓MAPK, 2.↑STAT1/MYC	AD	MSC-EVs in the AD context; source and species are not consistently specified across cited studies.	[155–160]
miR-21	M1/M2 (Dual function)	1.↑TLR7, ↑NF-κB 2.↓PDCD4, ↓PTEN, ↓NF-κB	TBI	HUC-MSC-EVs for the therapeutic arm and neuron-derived EVs for the injury-associated arm; rat TBI models.	[125–129]
miR-181b	↑M2	↓PTPN1, ↑IL-10/STAT3	TBI	BM-MSC-EVs; rodent TBI model.	[86, 130]
miR-125a	↑M2	↓IRF5	SCI	BM-MSC-EVs; rodent SCI model.	[133]
miR-133b	↓M1, ↑M2	↓RhoA, ↓MyD88	SCI	AD-MSC-EVs; rat SCI model.	[131, 134–136]
miR-24-3p	↓M1	1.↓MAPK9, ↓JNK/c-Jun/c-Fos. 2. ↓MK2.	SCI	BM-MSC-EVs; rodent SCI models.	[141, 142]
miR-21a-5p	↑M2	↓PELI1	SCI	BM-MSC-EVs; rodent SCI model.	[143–145]
miR-216a-5p	↓M1, ↑M2	1.↓HMGB1, ↓TLR4/NF-κB 2. ↑PI3K/AKT	SCI	MSC-EVs in SCI models; source and species are not consistently specified across cited studies.	[49, 70, 146, 147]
miR-155	↑M1	↓SOCS, ↓SHIP1, ↑JAK2/STAT1, ↑PI3K/Akt–NF-κB	AD	BM-MSC-EVs, including hypoxia-preconditioned MSC-EVs; rat SCI models.	[161–164]
miR-195	↑M2	1. ↓BACE1 2.↓CX3CL1/CX3CR1	AD	BM-MSC-EVs or microglia-associated miRNA contexts in AD; functional direction is context dependent.	[165, 166]
miR-140-3p	↓M1	1. ↓HMGB1 2.↓NLRP3/Caspase-1/GSDMD-N 3.↑S-Lactoylglutathione	SAE	EV/miRNA-based AD studies; source and species are not consistently specified across cited studies.	[172–174]
miR-494	↓M1, ↑M2	↓SIRT3	SAE	MSC-EVs; murine SAE models.	[175, 176]

↓: inhibit. ↑: promote. IS: ischemic stroke. TBI: traumatic brain injury. SCI: spinal cord injury. AD: Alzheimer’s disease. SAE: sepsis-associated encephalopathy

## Conclusion and future perspective

MSC-EVs offer a cell-free framework for modulating neuroinflammation, but their therapeutic interpretation should move beyond the general promise of EV therapy. Across the CNS disease models reviewed here, several regulatory nodes recur. TLR/NF-κB and MAPK signaling frequently mediate suppression of M1-like inflammatory activation, whereas PI3K/Akt, JAK/STAT and Wnt/β-catenin pathways contribute to reparative signaling, cell survival and microglial phenotype remodeling. miR-124/miR-124-3p, miR-146a-5p, miR-21/miR-21a-5p, miR-223-3p and miR-216a-5p appear repeatedly across inflammatory and injury contexts. These cargo-pathway axes may therefore provide useful starting points for mechanistic validation.

The evidence reviewed here also emphasizes context dependence rather than a universal miRNA program. The same miRNA may exert different effects according to disease stage, cellular source, EV donor type, recipient microglial state and pathway crosstalk. miR-21 and miR-155 illustrate this principle, as their functional direction depends on cellular origin and microenvironmental context. These observations support a network-based view in which MSC-EV-associated miRNAs tune interacting inflammatory, metabolic, pyroptotic and survival pathways, rather than simply switching microglia between fixed M1 and M2 states.

Clinical translation will therefore require more than scalable EV production. Future studies should standardize EV terminology and characterization, report MSC tissue source and donor species, and compare human and rodent MSC-EV preparations directly. They should also connect cargo profiles with potency assays that measure microglial responses. Particular attention should be paid to miRNA dose, off-target regulation, CNS biodistribution, delivery route, therapeutic window and long-term safety. A source-aware and species-aware framework will help identify the MSC-EV miRNA axes most suitable for reproducible, mechanism-guided development in CNS diseases.

## Data Availability

No datasets were generated or analysed during the current study.

## Abbreviations

Aβ: Amyloid beta
AD: Alzheimer’s disease
AD-MSC-EVs: Adipose-derived mesenchymal stem cell EVs
APP: Amyloid precursor protein
Arg-1: Arginase-1
BACE1: Beta-site APP cleaving enzyme 1
BBB: Blood-brain barrier
Bcl-2/Bax: B-cell lymphoma 2/Bcl-2-associated X protein
BM-MSC-EVs: Bone marrow mesenchymal stem cell-derived EVs
CNS: Central nervous system
CX3CL1/CX3CR1: C-X3-C motif chemokine ligand 1/C-X3-C chemokine receptor 1
CysLT2R-ERK1/2: Cysteinyl leukotriene receptor 2-extracellular signal-regulated kinases 1 and 2
DAMPs: Damage-associated molecular patterns
DAPK1: Death-associated protein kinase 1
FIP200: FAK family kinase-interacting protein of 200 kDa
GLT-1: Glutamate transporter 1
GSDMD: Gasdermin D
GSK-3β: Glycogen synthase kinase 3 beta
HMGB1: High-mobility group box 1
HUC-MSC-EVs: Human umbilical cord mesenchymal stem cell-derived EVs
I/R: Ischemia/reperfusion
IDE: Insulin-degrading enzyme
IGF-1: Insulin-like growth factor 1
ILs: Interleukins
iNOS: Inducible nitric oxide synthase
IRAK1: Interleukin-1 receptor-associated kinase 1
IS: Ischemic stroke
LPS: Lipopolysaccharides
MAPK: Mitogen-activated protein kinase
miRNAs: MicroRNAs
MK2: Mitogen-activated protein kinase-activated protein kinase 2
MSC-EVs: Mesenchymal stem cell-derived EVs
MSCs: Mesenchymal stem cells
mTOR: Mechanistic target of rapamycin
MyD88: Myeloid differentiation primary response 88
NEP: Neprilysin
NLRP3: NLR family pyrin domain containing 3
PAMPs: Pathogen-associated molecular patterns
PDCD4: Programmed cell death 4
PDE4B: Phosphodiesterase 4B
PELI1: Pellino E3 Ubiquitin Protein Ligase 1
PI3K/AKT: Phosphoinositide 3-kinase / protein kinase B
pro-IL-1β: Precursor interleukin-1 beta
PRRs: Pattern recognition receptors
PTEN: Phosphatase and tensin homolog
Rela/ApoE: RELA proto-oncogene/apolipoprotein E
ROCK1/ROCK2: Rho-associated coiled-coil containing protein kinase 1/2
ROS: Reactive oxygen species
S100A8/9: S100 calcium-binding protein A8 and A9
SAE: Sepsis-associated encephalopathy
SCI: Spinal cord injury
SHED-EVs: Stem cells from human exfoliated deciduous teeth-derived EVs
TBI: Traumatic brain injury
TGF-β: Transforming growth factor-beta
TLR4/NF-κB: Toll-like receptor 4/nuclear factor kappa-light-chain-enhancer of activated B cells
TNF-α: Tumor necrosis factor-alpha
TRAF6: Tumor necrosis factor receptor-associated factor 6
TREM1: Triggering receptor expressed on myeloid cells 1
Wnt/β-catenin: Wnt/beta-catenin signaling pathway
